# Extracellular Vesicles Derived from Human Umbilical Cord Perivascular Cells Improve Functional Recovery in Brain Ischemic Rat via the Inhibition of Apoptosis

**DOI:** 10.29252/ibj.24.6.342

**Published:** 2020-07-25

**Authors:** Elham Seifali, Gholamreza Hassanzadeh, Marzieh Mahdavipour, Keywan Mortezaee, Ashraf Moini, Leila Satarian, Faezeh Shekari, Abdoreza Nazari, Shabnam Movassaghi, Mohammad Akbari

**Affiliations:** 1 Department of Anatomy, School of Medicine, Tehran University of Medical Sciences, Tehran, Iran;; 2 Department of Anatomy, School of Medicine, Kurdistan University of Medical Sciences, Sanandaj, Iran;; 3 Department of Gynecology and Obstetrics, School of Medicine, Tehran University of Medical Science, Tehran, Iran;; 4 Department of Endocrinology and Female Infertility, Reproductive Biomedicine Research Center, Royan Institute for Reproductive Biomedicine, ACECR, Tehran, Iran;; 5 Breast Disease Research Center (BDRC), Tehran University of Medical Sciences, Tehran, Iran;; 6 Department of Brain and Cognitive Science, Cell Science Research Center, Royan Institute for Stem Cell Biology and Technology, ACECR, Tehran, Iran;; 7 Department of Stem Cells and Developmental Biology, Cell Science Research Center, Royan Institute for Stem Cell Biology and Technology, ACECR, Tehran, Iran;; 8 Department of Anatomy and cognitive neuroscience, School of Medicine, Tehran Medical Sciences Branch, Islamic Azad University, Tehran, Iran

**Keywords:** Apoptosis, Extracellular vesicles, Ischemia, Middle cerebral artery

## Abstract

**Background:**

Ischemic stroke, as a health problem caused by the reduced blood supply to the brain, can lead to the neuronal death. The number of reliable therapies for stroke is limited. MSCs exhibit therapeutic achievement. A major limitation of MSC application in cell therapy is the short survival span. MSCs affect target tissues through the secretion of many paracrine agents including EVs. This study aimed to investigate the effect of HUCPVCs-derived EVs on apoptosis, functional recovery, and neuroprotection.

**Methods:**

Ischemia was induced by MCAO in male Wistar rats. Animals were classified into sham, MCAO, MCAO + HUCPVC, and MCAO + EV groups. Treatments began at two hours after ischemia. Expressions of apoptotic-related proteins (BAX/BCl-2 and caspase-3 and -9), the amount of TUNEL-positive cells, neuronal density (MAP2), and dead neurons (Nissl staining) were assessed on day seven post MCAO.

**Results:**

Administration of EVs improved the sensorimotor function (*p < *0.001) and reduced the apoptotic rate of Bax/Bcl-2 ratio (*p < *0.001), as well as caspases and TUNEL-positive cells (*p *< 0.001) in comparison to the MCAO group. EV treatment also reduced the number of dead neurons and increased the number of MAP2^+^ cells in the IBZ (*p *< 0.001), as compared to the MCAO group.

**Conclusion:**

Our findings showed that HUCPVCs-derived EVs are more effective than their mother’s cells in improving neural function, possibly via the regulation of apoptosis in the ischemic rats. The strategy of cell-free extracts is, thus, helpful in removing the predicaments surrounding cell therapy in targeting brain diseases.

## INTRODUCTION

Stroke is a life-threatening cerebrovascular disease caused by the reduction of bloodstream to an area of the brain, leading to neurological failure and fatality^[^^1^^,^^2^^]^. Following cerebral ischemia, two zones are distinguishable in the brain. The core area experiences irreversible neuronal death^[^^3^^]^. The IBZ is at infarction risk; however, timely therapeutic intervention may prevent apoptosis and neuronal death^[^^4^^]^. Ischemia is characterized by a series of molecular events, including extreme production of free radicals and membrane depolarization that may lead to apoptosis in many brain cells^[^^3^^,^^5^^]^. 

Family members of the Bcl­2 regulate apoptosis by managing apoptotic signals^[^^6^^]^. Two members of this family are Bcl­2 and Bax. Bcl­2 is an anti-apoptotic protein that restrains the activity of Bax^[^^7^^]^. Bax is an essential apoptosis factor that promotes the activation of caspase enzymes, resulting in the activation of apoptosis and cellular disassembly in response to the early signals of apoptosis^[^^8^^]^. Caspase-mediated cell death may occur in many types of brain cells and cause neuronal injury^[^^9^^]^. 

In recent decades, MSCs have been used for repairing the central nervous system diseases in pre-clinical and clinical settings^[^^10^^,^^11^^]^. Most MSC effects are paracrine and may exert through the secretion of nano-sized particles, called EVs^[^^12^^,^^13^^]^. HUCPVCs have the potential for regeneration and protection of central nervous system^[^^14^^,^^15^^]^. Low homing rate and durability are considered the limitations of cell therapy. This approach can be modified using EVs that act as carriers of informational molecules and have adequate absorption, stability, and infinite storage capacity. Therefore, EVs as a tool for cell-free therapy have the potential of being more effective than their parent cells in adjusting the manner and action of the target cell^[^^16^^-^^18^^]^. EVs and their cell sources are reported to be effective against neuronal degeneration through the reduction of apoptosis^[^^19^^-^^21^^]^. As a rule, the use of EVs and their MSC sources have been established in the attenuation of apoptosis and reducing lesion area in myocardial ischemia, traumatic brain injury, and spinal cord injery^[^^22^^-^^24^^]^. 

The current investigation was designed assuming that EVs derived from HUCPVCs may also be effective for reducing apoptosis and improving functional rehabilitation in the rat model of MCAO brain ischemia.

## MATERIALS AND METHODS

**Animals handling and surgical procedure**

Adult healthy male Wistar rats (n = 24, 12 weeks old, weighing 270–300 g) were purchased from the Faculty of Pharmacy, Tehran University of Medical Sciences, Tehran, Iran. The rats were kept at a stable temperature of 22 °C, with a 12 h light/dark cycle. Food and water were provided *ad libitum*. Cerebral ischemia was induced by left MCAO as previously reported^[^^25^^,^^26^^]^. Briefly, rats were anesthetized with 5.0% isoflurane (Baxter Corporation, USA), while the left common, left external, and left internal carotid arteries were exposed to a ventral midline skin incision. A silicon-coated monofilament (Doccol Co., USA) was passed through common carotid artery to MCA. The monofilament was removed one hour after the induction of MCAO. Rats were divided into four groups (n = 6 per group): Sham, MCAO, MCAO + HUCPVCs, and MCAO + EVs. Animals in the sham group underwent the skin incision and dissection of arteries, but arterial occlusion was not performed. According to our pervious investigation^[^^27^^]^, at two hours after ischemia induction, the MCAO + HUCPVC and MCAO + EV groups received intra-cerebroventricular injection of HUCPVCs (3 × 10^5^ cells in 5 µl PBS) and EVs (50 µg of EVs in 5 µl PBS), respectively, into the left lateral ventricle with the following coordinates (from bregma: antro-posterior = -0.9 mm, medio-lateral = -1.8 mm (midline), and dorso-ventral= 3.5 mm deep from the dura)^[^^28^^]^.

**Cell culture**

***Isolation and culture of HUCPVCs***

HUCPVCs isolated from healthy human umbilical cords (gestational age = 39 weeks) were obtained from Arash Women’s Hospital (Tehran, Iran). The cells were collected according to previous protocols^[^^29^^]^. In brief, the dissected umbilical cord vessels were cut into 5-cm pieces. After incubation in 1 mg/ml of collagenase solution (Sigma-Aldrich, USA), the vessels were discarded and the solution was centrifuged (4000 ×g for 5 min). HUCPVCs were suspended in alpha-MEM medium (Invitrogen, USA) containing penestrep 1% (Gibco, USA) and 10% fetal bovine serum (Gibco) and plated in T75 flasks at a density of 3 × 10^3^ cells/cm^3^. Next, the flasks were transferred into a 5% CO_2_ incubator at 37 °C to reach 90% confluence. The multipotent behavior of the cells was confirmed by osteogenic and adipogenic differentiation using the respective Alizarin red and Oil red O staining^[^^30^^,^^31^^]^. To evaluate the osteogenic differentiation, third passaged HUCPVCs were cultured on six-well plates in the osteogenic medium (Gibco). After 21 days, the medium was removed, and cells were fixed in 4% formaldehyde solution for 30 min. They were then rinsed in distilled water and stained with 2% Alizarin Red solution (Gibco) for 4 min. Afterward, the wells were rinsed twice with distilled water and visualized under a light microscope. For adipogenic differentiation, third passaged cells were cultured on six-well plates in an adipogenic medium for 21 days. In the next step, they were washed with distilled water, fixed in 4% formaldehyde for 30 min and stained with 0.5% Oil red O solution (Gibco) at room temperature for 10 min. Eventually, Oil Red O solution was removed, and the samples were washed immediately with distilled water and observed under light microscopy.

**Flow cytometry**

HUCPVCs were incubated with FITC-conjugated monoclonal antibodies against CD90 and CD45 (eBioscience, Thermo Fisher Scientific, USA) and with PE-conjugated monoclonal antibodies against CD31 and CD146 (eBioscience, Thermo Fisher Scientific). Then the cells were washed and suspended in PBS (Gibco) and assessed under a BD FACSCalibur™ (BD Biosciences, USA). The FACS data were analyzed using FlowJo software (BD Bioscience).

**Isolation, purification, and characterization of HUCPVCs-EVs**

When the HUCPVCs reached 70-80% confluency, the culture medium was replaced with fetal bovine serum-free EV medium, and the conditioned medium was collected every 48 h from passages 3 to 5. The cell-free conditioned medium was centrifuged at 3000 ×g for 10 min to remove cell debris. Afterwards, EVs were isolated by three steps of differential centrifugation: 20,000×g for 30 min, followed by centrifugation of supernatant for 120 min at 110,000 ×g, subsequent resuspention of the pellet in 4 ml PBS (PBS lack of mg and Ca), and final centrifugation at 110,000×g for 120 min. The isolated EVs were characterized by SEM^[^^32^^]^. Moreover, the expression profiling of enriched EV proteins, including CD63 (1:500, ab8219), CD81 (1:500, sc-7637), and Calnexin (1:500, ab75801), was evaluated using Western blotting as described before^[^^33^^]^. Briefly, the EV proteins (30 μg) were first isolated, measured for protein concentration by Bradford assay (Bio-Rad, USA). The extracted proteins were run on a 12% SDS-PAGE, and transferred onto polyvinylidene fluoride membranes (Bio-Rad, France). The polyvinylidene fluoride membranes were blocked in Tris-buffered saline (Sigma-Aldrich) containing 5% powdered milk at 37 °C for 1 h and then incubated overnight with the primary antibodies at 4 °C. Next, samples were incubated with peroxidase-conjugated goat anti-rabbit secondary antibody (Bio-Rad, USA). The bands were visualized with an enhanced chemiluminescence system (ECL plus, Pierce Scientific, Waltham, MA, USA) and quantified using Total Lab Quant Analysis software (Total Lab Limited, England). The sizes of EVs were determined using a DLS device (Malvern Instruments, Malvern, UK).

**TTC staining**

TTC staining was performed to determine the infarct areas of the left cortex at 24 hours after MCAO. Animals were anesthetized with isoflurane, after which using a rat brain matrix, their brains were cut into 2-mm coronal sections and stained with 2% TTC (Sigma-Aldrich) solution for 10 min. Coronal sections were then extended from frontal to occipital areas and photographed using a digital camera (Olympus, Japan). The infarcted areas appeared in white^[^^34^^,^^35^^]^. 

**Neurobehavioral tests**

***Adhesive removal test***

The adhesive removal test was performed to assess somatosensory deﬁcit, adhering to previous guidelines^[^^36^^]^. Briefly, rats were familiarized with the test before starting the MCAO. After habituation to the cages, animals were transferred to the testing room and placed in a transparent box (36 × 19.5 × 18.5) for 5 min. Subsequently, they were taken out of the box and trained for three days to take off the yellow adhesive tapes (1 × 1 cm) pasted on each forepaw. Only animals able to remove the tapes in ≤10 s were selected for ischemia induction. The test was repeated at 1, 3, and 7 days after MCAO, and the time length of tape removal by the rats was recorded. 

***EBST***

 EBST is a locomotor reflexive behavior test that does not need training, as described by Borlongan *et al.*^[^^37^^]^. The rats were left in a transparent cage (40 × 40 × 35.5 cm). By clutching the tail, animals were raised up to 5 cm from the cage surface. The left or right motion was recorded when the head of the animals deviated at 10° to the sides from the midline. After the left MCAO, the rats swung mostly to the right. Control rats swung to the left or right in almost equal times, as previously documented^[^^38^^]^. After one swing, rats were placed in a supine position in the cages to move freely for 5 min. All steps were repeated 14 times for each animal. 

***IHC***

IHC staining was performed to evaluate the expressions of MAP2, Bax, and Bcl-2 at the protein level, as described earlier^[^^39^^]^. Briefly, brain coronal sections (10 µm) were deparaffinized. They were then incubated with a medium containing 0.3% Triton (Kiazist, Iran) and 10% goat serum (Capricorn Scientific GmbH, Germany), MAP2, Bax, or Bcl­2 primary antibodies (1:100; Abcam, USA) at 4 °C overnight. After washing twice with PBS, samples were incubated with rabbit anti-rat FITC-conjugated secondary antibody (Abcam) in the dark at 37 °C for 30 min. Samples were again washed with PBS, followed by adding DAPI (Sigma-Aldrich) to counterstain the nuclei. 

***Caspase-3 and -9 immunostaining***

Brain coronal sections (10 µm) were deparaffinized, placed in 1× TBS (Sigma-Aldrich) solution and boiled. After reaching the boiling temperature, the sections were removed and placed in the solvent (H_2_O_2_ and methanol mixture) for another 15 min. Samples were exposed to H_2_O_2_ and methanol solution for 10 min. Samples were then immersed in the blocking buffer (1% BSA containing 0.3% Triton-X-100) for 50 min, followed by incubation with caspase-3 and caspase-9 primary antibodies (1:100; Santa Cruz Biotechnology, Santa Cruz, CA). The sections were stored at 4 °C overnight. Then equal volumes (100 μl) of linker rabbit anti-rat IgG (Abcam, USA), polymer, and a chromogen DAB (Sigma-Aldrich) buffer were sequentially added to the samples for 20, 30, and 5 min, respectively. The slides were counterstained with hematoxylin and monitored by optical microscopy (Olympus). After each step, samples were washed with PBS. ImageJ software version 1.42q was used to analyze the images. Images were loaded onto the Image J software and then oxylin and eosin  DAB icon on the Image J software^[^^40^^]^. The brightness and contrast of the images were adjusted by calculating the density of brown color areas. Caspase-positive areas stained dark brown^[^^41^^]^. 

***TUNEL assay***

The in situ Cell Death Detection Kit (Roche Applied Science, Mannheim, Germany) was used to evaluate apoptosis. The 10-µm coronal sections were heated at 60 °C and washed using xylene, grading series of ethanol, and double-distilled water, in the order of their appearance. The sections were incubated with protease K at 37 °C for 30 min, then placed in 1% Triton X-100 for 10 min. After washing with PBS, the sections were stained with the TUNEL reaction mixture at 37 °C for ~1 hour. DAPI was used to counterstain the nuclei. The stained sections were washed, and apoptosis was calculated as the ratio of TUNEL-positive neurons to the total neurons. Counting was carried out twice, in a blinded fashion. Next, 10 fields from each slide (three sections for each animal) were randomly chosen and counted.

***Nissl staining***

On day seven after MCAO, rats were anesthetized and sequentially perfused with 150 ml of normal saline and 250 ml of 4% paraformaldehyde (Merck, Germany). Brain samples were removed and stored in 4% paraformaldehyde at 4 °C and then embedded in paraffin and sectioned. Brain coronal sections (10 µm) were deparaffinized in xylene with two changes for 10 min and hydrated in 100% alcohol twice for 5 min, 95% alcohol for 3 min, and 70% alcohol for 3 min. Sections were rinsed in tap water and distilled water and stained with 1% cresyl violet solution (Sigma-Aldrich) for 3-10 min^[^^42^^]^. 

**qRT-PCR**

The expression of the MAP2 gene was evaluated by qRT-PCR. Using TRIzol® Reagent (Qiagen, Germany), total RNA was collected from fresh cerebral tissues. Afterward, 1 µg of mRNA was converted to cDNA by reverse transcription using the First Strand cDNA Synthesis Kit (Fermentas, USA). For further analysis, primers, cDNA, and PCR reagents (polymerase, dNTP, magnesium, and buffer; 5× HOT FIREPol® EvaGreen® qPCR Mix Plus [ROX] 1 ml, Solis Bio Dyne, Estonia) were placed in the RT-PCR machine. Primer sequences of MAP2 and GAPDH primers were as follows: MAP2 forward (19 bp): CTT TTG TT GCT CGG GAT T and MAP2 reverse (20 bp): GGG TCA CTA AAC TGC CAC CT as well as GAPDH forward (22 bp): AAG TTC AAC GGC ACA GTC AAG G and GAPDH reverse (22 bp): CAT ACT CAG CAC CAG CAT CAC C. Samples were incubated at 95 °C for 15 min for initial polymerase activation. Then the following three steps were taken: denaturation, at 95 °C for 15 s; annealing, at 60 °C for 20 s; elongation, at 72 °C for 20 s. The ΔΔCt method was used for the relative quantification of data and normalization of GAPDH. The obtained data were represented as fold change mRNA expression compared to the sham group. 

**Statistical analysis**

Kolmogorov-Smirnov test was used to check the normal distribution of the obtained data. The entire data analysis was performed using a two-way analysis of variance (ANOVA), followed by the Tukey’s post hoc test. Results were expressed as mean ± SD, and comparisons were considered statistically significant at *p* values less than 0.05.

**Ethical statement**

The above-mentioned treatment/sampling protocols were approved by the Research Ethics Committee of Tehran University of Medical Science, Tehran, Iran (ethical code: IR.TUMS.MEDICINE. REC.1396.3189).

## RESULTS

**HUCPVC and** **HUCPVC-EV** **isolation and characterization**

**Fig. 1:**
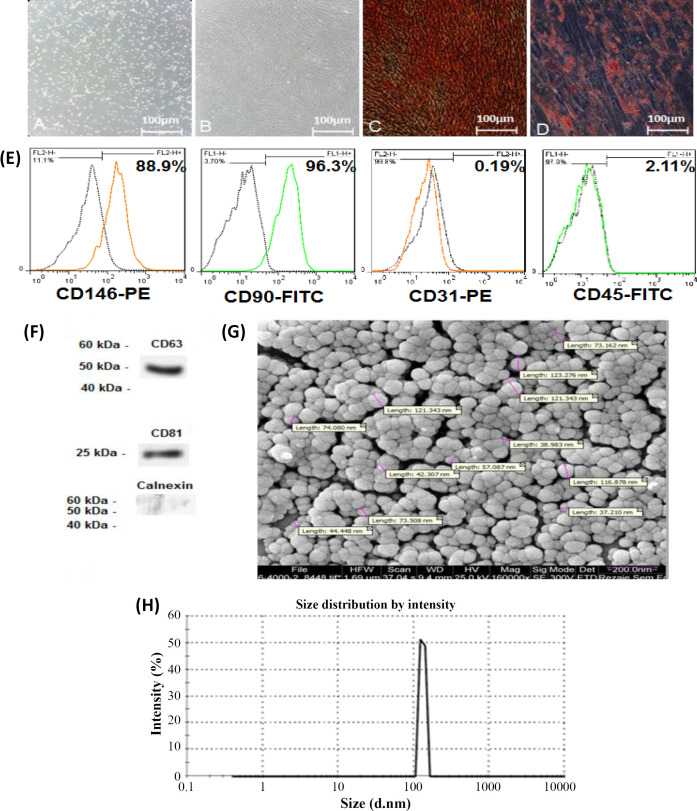
Characteristics of HUCPVCs-derived EVs. (A and B) HUCPVCs under routine cultivation conditions at passages 0 and 3 (×100 magnification); (C and D) multi-potential feature of the HUCPVCs, attested by the differentiation of the cells into osteogenic (Alizarin red staining) and adipogenic (Oil red O staining) lineages (×100); (E) flow cytometry for evaluating the expressions of cell surface markers in HUCPVCs; (F) Western blot results for the detection of protein expression of surface markers in EVs. EVs highly expressed CD63 and CD81, but Calnexin was not expressed in the particles; (G) SEM images showing that the HUCPVC-derived particles had spherical shape; (H) DLS histogram demonstrating that EVs had variable sizes ranging from 35-200 nm

After nine days of primary culture, the adherent cells exhibited a fibroblastic spindle-shape morphology and showed confluency and propensity to differentiate into osteogenic and adipogenic lineages (Fig. 1A-1D). According to the results of flow cytometry, HUCPVCs indicated a high rate of expression for MSC marker CD90 (96.3%) and pericyte marker CD146 (88.9%). Meanwhile, the cells were negative for hematopoietic cell marker CD45 (2.11%) and endothelial cell marker CD31 (0.19%), as represented in Figure 1E. Based on 

the Western blot results, HUCPVCs-EVs expressed CD63- and CD81-specific markers of EVs, while the cells were negative for Calnexin (Fig. 1F). The results of SEM (Fig. 1G) and DLS (Fig. 1H) demonstrated that the particles had spherical morphology (SEM outcomes) with a size range of 35-200 nm. EVs were revived from frozen stocks.

**TTC staining and neurobehavioral functions**

**Fig. 2:**
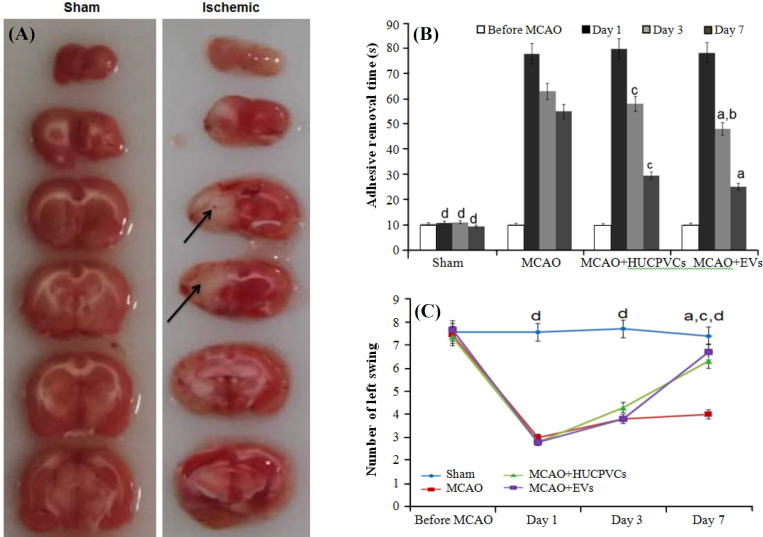
TTC staining of seven sequential coronal brain slices at 24 h after left MCAO and the effects of EVs derived from HUCPVCs on neurobehavioral functions. (A) Ischemic rats revealed white regions (arrows) in the left side of cortex; (B and C) results of the adhesive removal test and EBST at the 1^st^, 3^rd^, and 7^th^ days after MCAO. All data are shown as mean ± SD (ANOVA, n = six/group, and significant differences are indicated by lowercase letters (* p < *0.05; a, MCAO + EVs vs. MCAO; b, MCAO + EVs vs. MCAO + HUCPVCs; c, MCAO + HUCPVCs vs. MCAO; d, sham vs. all MCAO groups).

TTC staining was performed on samples from 24 h post MCAO induction, to confirm the MCAO model. The infarcted area in the left hemisphere cortex appeared in white (Fig. 2A), denoting the induction of ischemia, whereas in the sham-operated group, the cortex appeard in red.

**Adhesive removal test**

No notable differences existed between all the groups in the elimination of sticky tapes on the first day after MCAO of sticky tapes on the first day after MCAO (*p < *0.05). On day 3, however, the time spent for the removal of the adhesive tapes in the MCAO + EV group (48.11 ± 6.64) reduced significantly compared to the MCAO + HUCPVC (58.33 ± 5.26) and MCAO (63.00 ± 3.24) groups (*p < *0.001). On day seven post ischemia, the time reduced even more in the MCAO + EV (25.22 ± 3.15) and MCAO + HUCPVC (29.55 ± 2.83) groups compared to the MCAO group (*p < *0.001), indicating that the HUCPVCs EVs have positive effects on the improvement of sensorimotor impaired by ischemia. No significant differences found between EV-treated and HUCPVC-treated groups (Fig. 2B).

Assessment of asymmetrical motor and locomotor behavior 

There were no significant differences in the left swing between the HUCPVCs (4.3 ± 0.86) and EVs (3.8 ± 1.26) therapy groups compared to the MCAO group (3.7 ± 0.97; *p* < 0.05) on day three post ischemia. In contrast, a notable rise was in the left swing for the MCAO + EVs (6.7 ± 0.7) and MCAO + HUCPVC (6.3 ± 0.7) groups compared to the MCAO group (4.1 ± 1.05) on day seven post MCAO (*p < *0.001). However, the difference between the MCAO + EV and MCAO + HUCPVC groups was not statistically significant (Fig. 2C).

**Bax/** **Bcl­2** **ratio**

**Fig. 3:**
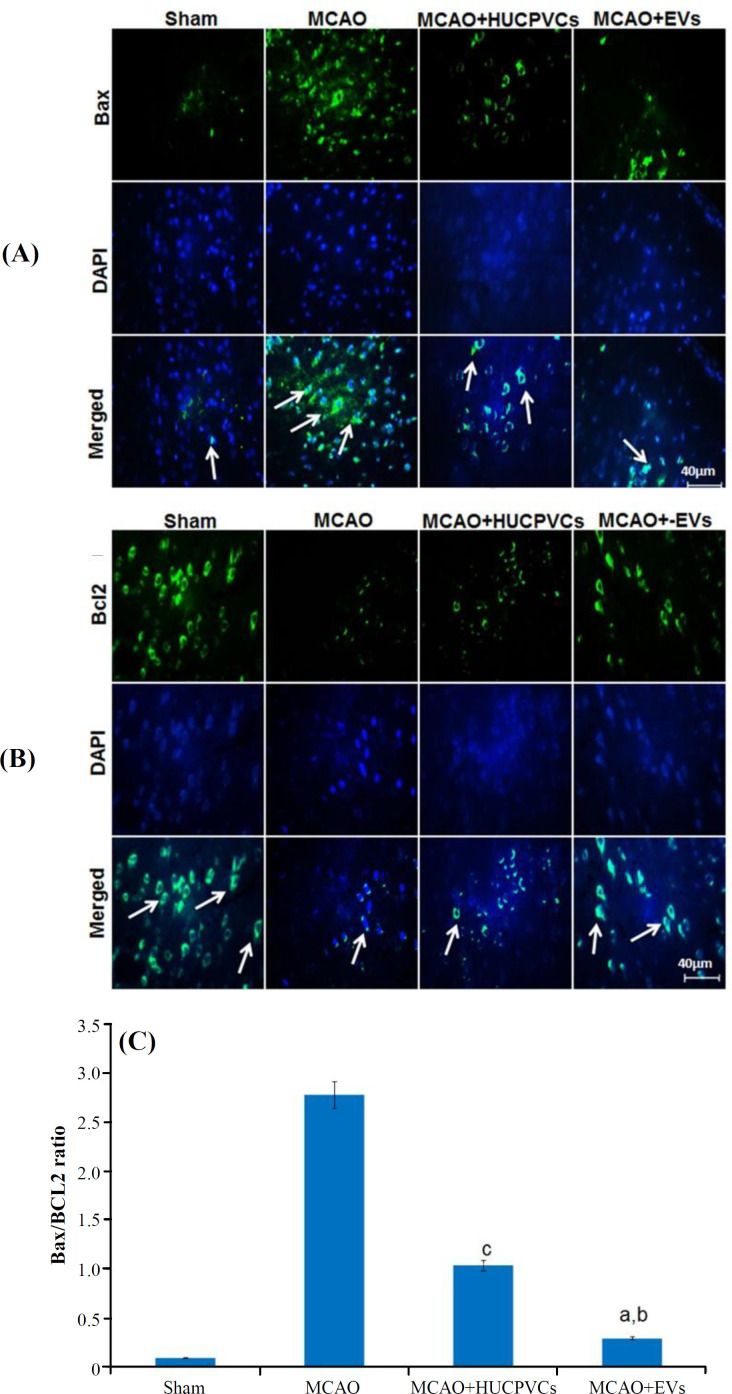
Effects of HUCPVC-EVs on Bax and Bcl­2 expression in the rat model of MCAO. The Figure shows qualitative and quantitative immunofluorescence outcomes. Arrows indicate the Bax and Bcl­2 positive cells. All data are represented as mean ± SD (ANOVA, n = 3/group). Significant differences are demonstrated by lowercase letters (*p < *0.05; a, MCAO + EVs vs. MCAO; b, MCAO + EVs vs. MCAO + HUCPVCs; c, MCAO + HUCPVCs vs. MCAO; d, sham vs. all MCAO groups).

IHC was performed to determine Bcl­2 and Bax protein expressions (Fig. 3). According to the quantitative results, the Bax/Bcl­2 ratio was reduced significantly in the EV- (*p < *0.001) and HUCPVC (*p < *0.05) treated groups, as compared to the MCAO group. Bax/Bcl-2 ratio in EV-treated group also reduced significantly in comparison to that of the HUCPVC-treated group (*p* < 0.05). 

**HUCPVCs-EVs effect on caspase-3 and -9 protein expression**

**Fig. 4:**
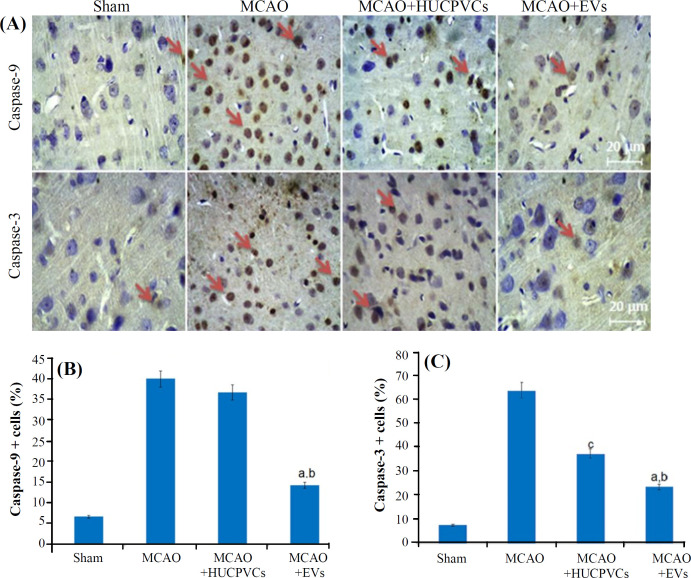
Caspase-9 and caspase-3 protein expressions measured after the administration of EVs derived from HUCPVCs in rats underwent MCAO ischemia induction. (A) IHC images of the caspase-9 and caspase-3. Arrows demonstrate the caspase-9 and caspase-3 positive cells. (B and C) Quantitative results for caspase 9 and 3, respectively, extracted from histological images using Image J software by measuring the intensity of brown staining areas. (n = 3/group, *p < *0.05). All data are presented as mean ± SD (ANOVA, *p < *0.05). Significant differences are shown by lowercase letters (*p < *0.05; a, MCAO + EVs vs. MCAO; b, MCAO + EVs vs. MCAO + HUCPVCs; c, MCAO + HUCPVCs vs. MCAO)

Caspase-3 (23.5 ± 3.33) and -9 (14.26 ± 1.54) expressions underwent a significant reduction in the EV-treated group, as compared to the MCAO + HUCPVCs (caspase-3, 37.41 ± 1.68 and caspase-9, 36.75 ± 2.48; *p* < 0.01) as well as MCAO (caspase-3, 64 ± 13.49 and caspase-9, 40 ± 7.07; *p* < 0.001) groups, evaluated at day seven post MCAO. The expression of caspase-3 also decreased in the HUCPVC-treated group, compared to the MCAO group (*p* < 0.001; (Fig. 4). 

**TUNEL** **staining**

**Fig. 5:**
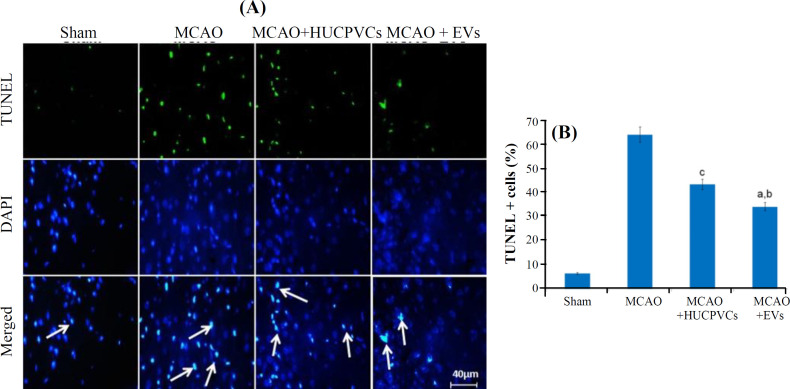
TUNEL assay used for the detection of apoptosis after EV administration to the rats underwent MCAO ischemia induction. (A) TUNEL-labeled cells (marked by arrows) at day seven after MCAO; (B) quantification and statistical analysis of the TUNEL-positive cells. Data are shown as mean ± SD (ANOVA, *p < *0.05; n = 3/group). Significant differences are presented by lowercase letters (*p* < 0.001; a, MCAO + EVs vs. MCAO; b, MCAO + EVs vs. MCAO + HUCPVCs; c, MCAO + HUCPVCs vs. MCAO)

TUNEL is a technique used for detecting apoptotic cells in histological sections. The amount of TUNEL positive cells was markedly raised in all test groups (63 ± 4.1), which underwent ischemia, in reference to the sham-treated group (6.5 ± 0.5; *p < *0.001). In the MCAO + EV group (34.3 ± 0.57), The proportion of the apoptotic cells to the total cells was decreased significantly compared to the MCAO (64.5 ± 3.27) and MCAO + HUCPVC (43.6 ± 3.05) groups in the IBZ (for both *p < *0.001). Meanwhile, in the HUCPVC-treated group, the quantity of TUNEL positive cells was significantly reduced compared to the MCAO group (*p < *0.001; Fig. 5). 

**Effects of HUCPVCs-EVs on dead neurons in the IBZ**

**Fig. 6:**
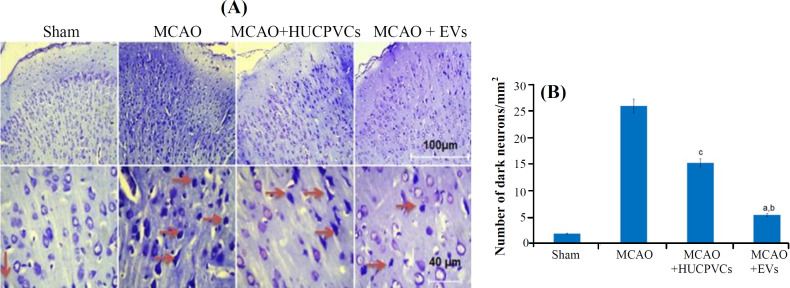
Effects of EVs on viability of neurons in the rat model of cerebral ischemia induced by MCAO. (A-B) Histological images and quantitative results obtained from Nissl staining using Image J software by counting the number of dark neurons. Data are shown as mean ± SD (ANOVA, *p < *0.05; n = 3/group). Significant differences are presented by lowercase letters (*p < *0.05; a, MCAO + EVs vs. MCAO; b, MCAO + EVs vs. MCAO + HUCPVCs; c, MCAO + HUCPVCs vs. MCAO).

Nissl staining was assessed to evaluate the number of dead neurons. Based on the obtained results, the MCAO + EV group (5.3 ± 1.75) had a significant reduction in the number of dead neurons compared to the MCAO + HUCPVCs (15.16 ± 3.3) and MCAO (25.83 ± 4.62) groups (for both *p < *0.001; Fig. 6).

**The effects of HUCPVC-EVs on neuronal density in the IBZ**

**Fig. 7:**
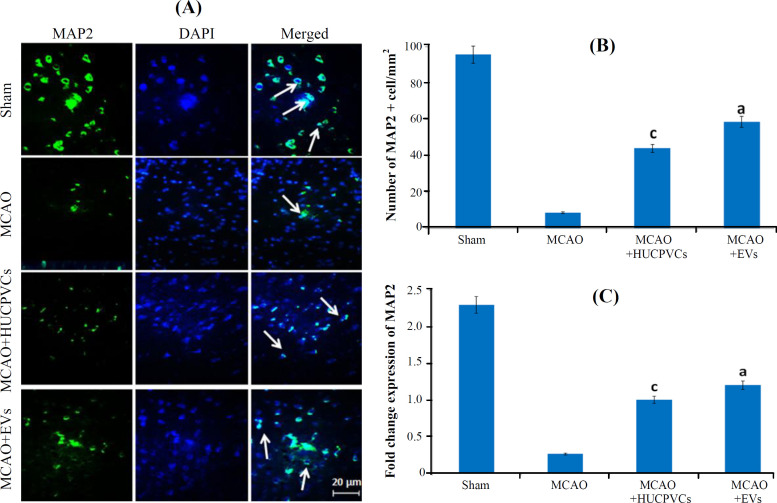
Effects of EVs on the expression of MAP2 gene and protein expression in rats underwent MCAO ischemia induction. (A and B) results from IHC; (C) qRT-PCR findings. Data are shown as mean ± SD (ANOVA, *p < *0.05; n = 3/group). Significant differences are represented by lowercase letters (*p* < 0.05; a, MCAO + EVs vs. MCAO; b, MCAO + EVs vs. MCAO + HUCPVCs; c, MCAO + HUCPVCs vs. MCAO; d, sham vs. all MCAO groups)

Data from Figure 7 show the findings for MAP2 expression in the studied groups. The EV-treated rats had a meaningful rise in the amount of MAP2^+^ neurons (59.5 ± 3.72) compared to the MCAO (Fig. 7A and 7B; *p < *0.001). The MAP2 gene expression exhibited a 4.6fold increase in the EV-treated group (1.2 ± 0.77) compared to the MCAO group (0.26 ± 0.073; *p < *0.001; Fig. 7C). 

## DISCUSSION

This research aimed to investigate the impacts of HUCPVCs-EVs on functional neuronal recovery, prevention of further damage, and neuronal apoptosis associated with ischemia. Our outcomes showed that EVs therapy after MCAO decreased neuronal apoptosis, increased neuronal density, reduced dark neurons and the infarct size and improved the sensorimotor function. Ample evidence exists on the efficacy of cell-based therapy for stroke^[^^43^^,^^44^^]^. MSCs-EVs are assumed as the main paracrine effects of MSCs and a reliable supersede with lower oncological risks^[^^45^^]^. Our results also support the studies elucidating that EVs secreted by MSCs provide a mechanism for participation in cellular communication with other cells, as well as protein and RNA transfer for tissue repair^[^^46^^,^^47^^]^. 

The present study disclosed that HUCPVCs-EVs improved sensory and motor performance in the ischemic rats. Xin *et al.*^[^^48^^]^ demonstrated that EVs derived from bone marrow MSCs improve neurological function and neuronal regeneration in the animal model of ischemia. These outcomes are consistent with the indirect effects of MSCs, emphasizing that MSCs are likely to exert their paracrine effects via the secretion of the EVs^[^^49^^]^. These vesicles are included in cell synergy, signaling, and adjusting cell metabolism and consequence tissue metabolism in the body^[^^50^^]^. Rare homing and endurance and tumorigenicity are barriers to cell therapy. This approach can be modified using EVs that are nanosize vesicles and carriers of informational molecules having sufficient absorption, establishment, and unlimited storage capacity^[^^20^^]^. Improvement of sensorimotor function after cell therapy and their cell-free extracts, namely EVs, is related to neurogenesis, neovascularization, and restraint of apoptosis and increase the livability of cerebral cells after MCAO^[^^51^^-^^53^^]^.

Apoptosis plays a pivotal role in neuronal death after stroke^[^^3^^,^^54^^]^. Apoptotic cells are degraded in an organized way to minimize the suffering and disruption of neighbor cells^[^^55^^]^. Joerger-Messerli *et al.*^[^^20^^]^ showed that EVs derived from Wharton’s jelly MSCs reduced apoptosis induced by perinatal hypoxia-ischemia. The results of our research revealed that the HUCPVCs-EVs promoted their neuroprotective effects through the attenuation of apoptosis. We realized that the quantity of TUNEL influenced cells decreased significantly in the EVs-treated group. Concurrent with the reduction of TUNEL-positive cells in the MCAO + EV group, down-regulation of Bax protein and up-regulation of Bcl­2 protein were detected in the present study. This result is in concordance with the study carried out by Huang *et al.*^[^^24^^]^ who found that the MSCs-EVs administration unregulated Bcl­2 in SCI rats. The Bcl-2 family proteins (Bax and Bcl­2) control and regulate the intrinsic (or mitochondrial) pathway of apoptosis^[^^56^^]^. By localizing in the mitochondrial outer membrane, Bcl-2 protein plays an essential role in the cell survival via the inhibition of pro-apoptotic Bax protein^[^^57^^]^ and through the increased Bax/Bcl-2 ratio at different times after ischemia/reperfusion injury predisposes neural cells to apoptosis^[^^58^^,^^59^^]^. Complementary and similar to these findings, evaluation of caspase-3 and -9 proteins revealed a noticeable decrease in the ischemic rats receiving EVs. Caspases are proteases associated with the process of programmed cell death^[^^60^^]^. Similarly, results of our research exhibited a meaningful diminution in the number of dark neurons for the EVs-treated rats, suggesting the protective effect of this therapeutic approach in improving neuronal recovery and reduction of neuronal cell death; this result is in line with the findings of other studies reported for SCI^[^^61^^,^^62^^]^. Finally, studying MAP2 gene and protein expression in the IBZ displayed a raise in the EVs-treated group in comparison with the MCAO. EVs and secretome of MSCs are reported to increase the density of MAP2^+^ mature neurons inside the body and laboratory environment^[^^14^^,^^23^^]^, which is possibly associated with the rebuilding of injured neurons in the IBZ^[^^63^^]^.

Overall, it is reasonable to assert that EVs are possibly appropriate candidates for regulating apoptosis and improving neuronal functional recovery in the ischemic brain. Furthermore, it can be stated that this cell-free approach is more effective than cell therapy for such purposes. The results of this study provide evidence favoring the efficacy of the application of EVs derived from HUCPVCs for improving sensorimotor function in the ischemic rats, possibly through the regulation of apoptosis. This strategy could benefit targeting brain diseases, using cell-free extracts in the clinic.

## CONFLICT OF INTEREST.

None declared.
